# Advances in research on pharmacotherapy of sarcopenia

**DOI:** 10.1002/agm2.12168

**Published:** 2021-08-09

**Authors:** Yang Feike, Liu Zhijie, Chen Wei

**Affiliations:** ^1^ Department of Geriatric Medicine The Central Hospital of Changsha City Changsha China

**Keywords:** aging, muscle wasting, pathogenesis, pharmacotherapy, sarcopenia, signaling

## Abstract

Sarcopenia is a comprehensive degenerative disease with the progressive loss of skeletal muscle mass with age, accompanied by the loss of muscle strength and muscle dysfunction. As a new type of senile syndrome, sarcopenia seriously threatens the health of the elderly. The first‐line treatment for sarcopenia is exercise and nutritional supplements. However, pharmacotherapy will provide more reliable and sustainable interventions in geriatric medicine. Clinical trials of new drugs targeting multiple molecules are ongoing. This article focuses on the latest progress in pharmacotherapeutic approaches of sarcopenia in recent years by comprehensively reviewing the clinical outcomes of the existing and emerging pharmacotherapies as well as the molecular mechanisms underlying their therapeutic benefits and side effects.

## Graphical abstract

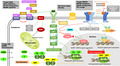

## 1 INTRODUCTION

**FIGURE 1:**
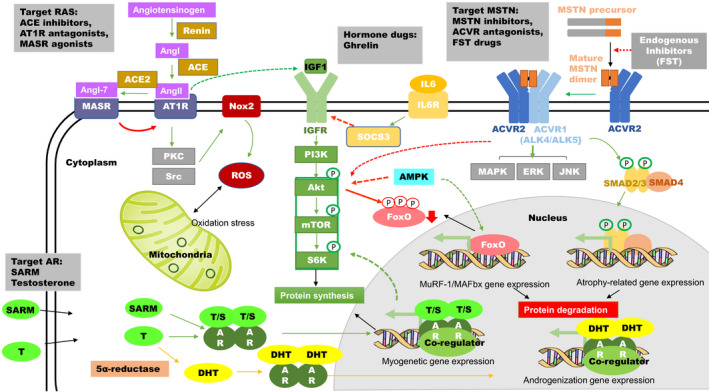
Signaling of the pharmacotherapies of sarcopenia. By targeting multiple pathways, such as myostatin (MSTN), renin‐angiotensin system (RAS), androgen receptor (AR), activated protein kinase (AMPK) signaling, potential drugs rebalance protein synthesis and degradation, reshape the endocrine system, reduce oxidative stress and promote mitochondrial function, result in beneficial effects in muscle hypertrophy. Green and red arrows demonstrate promoting and inhibiting effects, respectively. Yellow arrows indicate the pathway of side effect. The indirect impacts are illustrated with dash lines.

Sarcopenia is a comprehensive degenerative disease with the progressive loss of skeletal muscle mass with age, accompanied by the loss of muscle strength and muscle dysfunction. As a kind of senile disease, sarcopenia seriously affects the health and quality of life of the elderly. Patients with sarcopenia have reduced muscle function, restricted mobility, are prone to falls and fractures, and may induce diabetes and other chronic non‐communicable diseases, and even increase the risk of death. How to better prevent and treat sarcopenia has become one of the frontiers of global geriatric research. Over the last decade, there has been a remarkable increase in the understanding of the molecular mechanism of the pathogenesis of sarcopenia. For example, the role of cell signaling regulating protein synthesis and degradation in sarcopenia onset and development has been illustrated. Many molecules have been identified as therapeutic targets, bringing a booming of drug investigation targeting this disease. Here, we will focus on the molecular mechanisms (Figure 1) and the preclinical and clinical approaches (Table 1) of existing and emerging pharmacotherapies designed or repurposed for treating sarcopenia.

**TABLE 1:** The emerging drugs processed in clinical trials

Drug names	Type	Trial number	Phase	Trial start date completion date (or estimated completion date)	Trial result released or not	Trial title	Reference
Landogrozumab (LY‐2495655)	MSTN inhibitor	NCT01604408	2	2012‐2013	Yes	A Study in Older Participants Who Have Fallen and Have Muscle Weakness	^10^
Trevogrumab (REGN‐1033)	MSTN inhibitor	NCT01963598	2	2013‐2015	No	Study of the Safety and Efficacy of REGN1033 (SAR391786) in Patients With Sarcopenia	NA
Bimagrumab (BYM‐338)	Activin receptor antagonists	NCT01601600	2	2012‐2013	Yes	A Multi‐center Study to Assess the Effects of BYM338 on Skeletal Muscle in Sarcopenic Adults	^15^
		NCT02333331	2	2014‐2018	Yes	Dose Range Finding Study of Bimagrumab in Sarcopenia	^16^
		NCT02468674	2	2015‐2018	Yes	A 24‐week Off‐drug Extension Study in Sarcopenic Elderly Who Completed Treatment in the 6‐month Core Study	^16^
		NCT0192520	2/3	2013‐2016	Yes	Efficacy and Safety of Bimagrumab/BYM338 at 52 Weeks on Physical Function, Muscle Strength, Mobility in sIBM Patients (RESILIENT)	^17^
Bimagrumab (BYM‐338)	Activin receptor antagonists	NCT01669174	2	2012‐2014	Yes	BYM338 in Chronic Obstructive Pulmonary Disease (COPD) Patients With Cachexia	^18^
		NCT02152761	2	2014‐2018	Yes	Study of Efficacy and Safety of Bimagrumab in Patients After Hip Fracture Surgery	NA
Ramatercept (ACE‐031)	Activin receptor antagonists	NCT00952887	1	2009‐2011	Yes	A Safety, Tolerability, Pharmacokinetic and Pharmacodynamic Study of ACE‐031 in Healthy Postmenopausal Women	^19^
		NCT01099761	2	2010‐2011	Yes	Study of ACE‐031 in Subjects with Duchenne Muscular Dystrophy	^20^
ACE‐2494	Activin receptor antagonists	NCT03478319	1	2018‐2019	NA	A Study of ACE‐2494 in Healthy Subjects	NA
ACE‐083	Follistatin fusion proteins	NCT02257489	1	2014‐2016	Yes	Phase 1 Study of ACE‐083 in Healthy Subjects	^27^
		NCT02927080	2	2016‐teminated in 2019	Yes	Study of ACE‐083 in Patients with Facioscapulohumeral Muscular Dystrophy (FSHD)	NA
		NCT03124459	2	2017‐teminated in 2020	Yes	Study of ACE‐083 in Patients with Charcot‐Marie‐Tooth Disease	NA
rAAV1.CMV.huFollistatin 344	Follistatin gene therapy	NCT01519349	2	2012‐2017	Yes	Follistatin Gene Transfer to Patients With Becker Muscular Dystrophy and Sporadic Inclusion Body Myositis	31, 32
		NCT02354781	1/2	2015‐2017	Yes	Clinical Intramuscular Gene Transfer of rAAV1.CMV.huFollistatin344 Trial to Patients With Duchenne Muscular Dystrophy	NA
Perindopril (and /or Leucine)	ACE inhibitor	EudraCT # 2014‐003455‐61	NA	2015‐2021	No	Perindopril and Leucine to improve muscle function in older people	^39^
Perindopril	ACE inhibitor	NCT01891513	NA	2014‐2017	No	ACE Inhibitors Combined With Exercise for Seniors – Pilot Study (ACES‐P)	^40^
		NCT03295734	2	2019‐2023	No	ACES – ACE Inhibitors Combined With Exercise for Seniors With Hypertension (ACES)	^41^
Losartan	AT1 receptor antagonists	NCT02676466	2	2016‐2018	Yes	The ENRGISE (ENabling Reduction of Low‐Grade Inflammation in SEniors) Pilot Study (ENRGISE)	^44^
		NCT01989793	2	2013‐2016	Yes	A Study of Muscle Strength Maintenance in Older Adults	NA
BIO101	MASR agonists	NCT03452488	2	2018‐2021	No	A Double‐blind, Placebo Controlled, Randomized INTerventional Clinical Trial (SARA‐INT) (SARA‐INT)	NA
Testosterone	Testosterone	NCT00240981	4	2005‐teminated in 2009	Yes	TOM: Testosterone in Older Men With Sarcopenia	^51^
		NCT00799617	3	2009‐2014	Yes	The Testosterone Trials in Older Men	^52^
		NCT00190060	4	2004‐2008	Yes	Study of The Effects of Testosterone in Frail Elderly Men	^58^
		NCT00104572	2	2004‐2015	Yes	Effects of Aromatase Inhibition Versus Testosterone in Older Men With Low Testosterone: Randomized‐Controlled Trial.	53, 54
Testosterone, Finasteride	Testosterone, 5α‐reductase inhibitor	NCT00475501	2	2007‐2014	Yes	5‐Alpha Reductase and Anabolic Effects of Testosterone	^57^
Testosterone, supervised exercise training, home exercise program, health education modules	Testosterone, exercise	NCT02938923	3	2017‐2022	No	Starting a Testosterone and Exercise Program After Hip Injury (STEP‐HI)	NA
Sustanon 250, Zoladex	Testosterone, a sex hormone inhibitor	NCT03054168	3	2016‐unknown	No	Effects of Transdermal Testosterone and/or Monthly Vitamin D on Fall Risk in Pre‐‐frail Hypogonadal Seniors (T&D)	NA
MK‐0773	SARM	NCT00529659	2	2017‐2019	Yes	A Study of the Safety and Efficacy of MK‐0773 in Women With Sarcopenia (Loss of Muscle Mass) (MK‐0773‐005)	^60^
GTx‐024	SARM	NCT01355497	3	2011‐2014	Yes	Effect of GTx‐024 on Muscle Wasting in Patients With Non‐Small Cell Lung Cancer (NSCLC) on First Line Platinum	62, 63
		NCT01355484				Phase III Study of the Effect of GTx‐024 on Muscle Wasting in Patients With Non‐Small Cell Lung Cancer (NSCLC)	
GSK2881078	SARM	NCT03359473	2	2018‐2019	Yes	Study to Evaluate the Safety and Efficacy of 13 Weeks of the Selective Androgen Receptor Modulator (SARM) GSK2881078 in Chronic Obstructive Pulmonary Disease (COPD)	NA
CP‐424,391 (Capromorelin)	Ghrelin mimetics	NCT00527046	2	1999‐2001	Yes	Effects Of An Oral Growth Hormone Secretagogue In Older Functionally Limited Adults	^70^
Anamorelin	Ghrelin mimetics	NCT01387269	3	2011‐2015	Yes	Safety and Efficacy of Anamorelin HCl in Patients With Non‐Small Cell Lung Cancer‐Cachexia (ROMANA 1)	^72^
		NCT01387282				Safety and Efficacy of Anamorelin HCl in Patients With Non‐Small Cell Lung Cancer‐Cachexia (ROMANA 2)	
Metformin	AMPK activator	NCT02308228	1	2015‐2018	Yes	Metformin to Augment Strength Training Effective Response in Seniors (MASTERS)	^80^
		NCT01804049	1/2	2014‐2018	Yes	Metformin and Muscle in Insulin‐resistant Older Veterans (M&M)	NA
		NCT02570672	2	2016‐2024	No	Metformin for Preventing Frailty in High‐risk Older Adults	NA
		NCT03309007	3	2016‐2019	Yes	A Double‐Blind, Placebo‐Controlled Trial of Anti‐Aging, Pro‐Autophagy Effects of Metformin in Adults With Prediabetes	NA

ACE, angiotensin‐converting enzyme; AMPK, activated protein kinase; MASR, mitochondrial assembly receptor; MSTN, myostatin; SARM, selective androgen receptor modulator.

## 2 DRUGS TARGETING MYOSTATIN SIGNALING

Myostatin (MSTN), also known as growth differentiation factor‐8 (GDF‐8), belongs to the transforming growth factor β (TGF‐β) superfamily and functions as a negative regulator of muscle mass.^1^, ^2^ As a secreted hormone, it binds to activin type 2 receptors (ACVR2s) on muscle fiber membranes, which recruits and activates activin type I receptor‐like kinases, ALK4 and ALK5 (also known as ACVR1B and TGFBR1) to phosphorylate SMAD2 and SMAD3. Phosphorylated SMAD2 and SMAD3 form a complex with SMAD4, which consequently translocates into nuclear to promote atrophy‐related gene expression.^3^ Moreover, activated ALK4 and ALK5 can also impact some SMAD‐independent pathways, such as ERK, JNK, and p38 MAPK to regulate muscle growth, proliferation, and differentiation.^4^ At the same time, MSTN inhibits muscle growth by reducing Akt signaling^5^: on the one hand, it inhibits protein syntheses regulated by Akt/mTOR pathway; on the other hand, it promotes protein degradation by releasing the suppression of the FoxO pathway. The MSTN pathway is further regulated by the MSTN mature process and extracellular binding proteins, such as follistatin that are typically thought to function as antagonists (Figure 1).

Besides biochemical and cell biological evidence, the importance of MSTN signaling in muscles is further confirmed in genetic studies. The polymorphisms of genes involved in the pathway, including MSTN, ACVR2B, ACVR1B, and follistatin, are highly associated with individual variation for muscle strength and some muscle diseases, reviewed in ref. 6 In the mouse model, gene knockout or mutation of MSTN can cause muscle hypertrophy, whereas the increase of MSTN level will induce muscle atrophy.^7^ Moreover, the MSTN inhibitor, PF‐354, can effectively increase muscle mass and muscle function in mice.^8^

The inhibition of the MSTN pathway has become an attractive therapeutic strategy for stimulating muscle growth and/or preventing muscle wasting in many muscle‐wasting diseases, including sarcopenia, comprehensively reviewed in ref. 2. There are several types of drugs targeting this pathway progress in clinical trials, including MSTN inhibitors, activin receptor antagonists, and follistatin‐based drugs. However, some of these drugs also impact the activities of other closely related TGF‐β family members, such as GDF11, activins, and bone morphogenetic proteins (BMPs), which potentially bring unwanted side effects. For example, follistatin (FST), an endogenous antagonist of myostatin, can block both MSTN and GDF11 interacting with ACVR2B. Overexpression of FST increases skeletal muscle mass by suppressing the activity of myostatin, but diminishes BMP and induces bone fractures likely through repressing the activity of GDF11.^9^ Therefore, we need to carefully distinguish the molecular activities and adverse clinical effects of these drugs. In the following sections, we will focus on the clinical trials in sarcopenia, as well as other muscle‐wasting diseases whose data provide some transferable information for future sarcopenia studies.

### 2.1 MSTN inhibitor

Landogrozumab (LY‐2495655) is a humanized monoclonal antibody targeting myostatin. In its phase II trial in patients with sarcopenia (NCT01604408, completed in 2013), landogrozumab treatment significantly increased muscle mass and partially improved muscle function and mobility of older people aged 75 years or over.^10^ The treatment continued for 20 weeks with a 315 mg of landogrozumab injection every 4 weeks, followed by 16 weeks of observation. Interestingly, the treatment only significantly improved some physical performances measured by stair climbing time, the ability of hand rise with arms, fast gait speed, but not others measured by usual gait speed, 6‐minute walking distance, handgrip strength, and isometric leg extension strength.

Trevogrumab (REGN‐1033) is another monoclonal anti‐myostatin antibody, which specifically binds myostatin but not GDF11 or activin, and has effectively increased muscle mass and improved isometric force production in a mouse model.^11^ Its phase II clinic trial targeting sarcopenia (NCT01963598, completed in 2015) was completed in 2015, but the data evaluation is still underway.

Several other MSTN inhibitors have progressed to clinical trials for muscle‐wasting diseases, compressively reviewed in ref. 2 including monoclonal antibodies (Stamulumab [Myo‐29], Domagrozumab [PF‐06252616], and SRK‐015), a novel anti‐myostatin peptide PINTA‐745 (AMG‐745), and an anti‐myostatin adnectin RG6206 (RO7239361/BMS‐986089). They all only bind to MSTN instead of other TGF‐β family members and have shown promising effects on muscle hypertrophy with good safety profiles in preclinic studies. Unfortunately, except for SRK‐015, they all fail in their clinical trials due to the lack of efficiency to either increase muscle mass or improve muscle function. SRK‐015 is currently being investigated in a phase II trial in patients with spinal muscular atrophy.

The drug development of MSTN inhibitors is thriving in recent 10 years. Although there are frustrating fails of many candidates with highlight profiles in preclinic studies, new potential candidates, such as small molecule compound IMB0901,^12^ continue to be developed to bring new hope.

### 2.2 Activin receptor antagonists

Like MSTN inhibitors, the antagonists of MSTN’s receptor ACVR2s are capable of blocking the interaction between MSTN and ACVR2, therefore it inhibits MSTN induced muscle atrophy. For example, bimagrumab (BYM‐338), a due specific monoclonal antibody developed by Novartis Institutes for BioMedical Research, binds both to ACVR2A and ACVR2B with a greater affinity for ACVR2B than ACVR2A.^13^ Partial blockage of the pathway with either single receptor only results in a small increase in muscle mass. Bimagrumab, with its due specificity, fully blocked activation of ACVR2s induced by MSTN, significantly increases muscle mass in the mouse model.^13^, ^14^ In a proof‐of‐concept study with a phase II clinical trial (NCT01601600, competed in 2013) conducted in patients with sarcopenia, 16‐week treatment with 30 mg/kg of bimagrumab increased thing muscle mass and grip strength and improved mobility in those with slow walking speed.^15^ The promising result promoted conducting a large global clinic trial with 1221 individuals screened at 58 sites to confirm and better define the clinical benefit of bimagrumab treatment in sarcopenia. In this phase II/III trial (NCT02333331, completed in 2018), 220 patients were randomized after screening and divided into a placebo group and the other three drug groups to investigate dose range (6 monthly doses of 70, 210, or 700 mg). All patients received standard care with adequate nutrition and light exercise. An accompany trial (NCT02468674, completed in 2018) conducted a 24‐week off‐drug extension study followed after 6‐month treatment. These 4‐year trials were completed in 2018 and the data analysis and evaluation are currently underway. A recently published result compared the high dose group (700 mg) with the placebo group and showed bimagrumab significantly increased lean body mass and decreased fat body mass with good safety and tolerance. Both treatment and placebo groups showed an improvement in muscle physical function, however, there is no significant difference between the two groups.^16^ Bimagrumab has been also tested in other muscle‐wasting conditions, such as sporadic inclusion body myositis (sIBM; NCT01925209, completed in 2016),^17^ chronic obstructive pulmonary disease (COPD; NCT01669174, completed in 2014),^18^ muscle wasting after hip fracture surgery (NCT02152761, completed in 2018), etc. From current evidence, bimagrumab has shown consistent clinic benefit in increasing muscle mass, but not in improving muscle strength and function.

Soluble forms of ACVR2, such as Ramatercept (ACE‐031), can also function as antagonists by competitively binding ACVR2’s ligands, including MSTN. Ramatercept, developed by Acceleron Pharma for treating Duchenne muscular dystrophy (DMD), significantly increased the cross‐sectional area of both types I and II muscle fiber in a mice model^19^ and phase I trial (NCT00952887, completed in 2011) in healthy postmenopausal women.^20^ However, its phase II trial (NCT01099761, terminated in 2011) was terminated due to serious non‐muscle‐related adverse events, such as telangiectasia, nose‐bleed, gum‐bleed, and/or erythema.^21^ ACE‐2494, an improved version of ACE‐031, effectively stimulated muscle mass, bone length, and diaphyseal bone growth in a mouse model.^22^ Although ACE‐2494 showed promising early signs of target engagement in a phase I trial (NCT03478319, terminated in 2019), Acceleron Pharma announced their plan to discontinue the study due to the frequency of anti‐drug antibodies (ADAs) observed among participants.

### 2.3 Follistatin fusion proteins and gene therapy

Follistatin has at least six isoforms generated through alternative gene splicing and post‐translational proteolysis. These different isoforms have different bioactivities to bind and regulate the aforementioned TGFβ superfamily ligands. Follistatin isoforms all contain a heparin‐binding site (HBS), which is responsible for binding the heparin/heparin sulfate group existing in glycoproteins on the cell surface as well as in the extracellular matrix. Because the intermolecular interaction between the C‐terminal tail and HBS blocks HBS to bind heparin/heparin sulfate, the cleavage of the C‐terminal tail during post‐translational proteolysis disrupts the intermolecular interaction resulting in generating isoforms with higher affinities for cell surface/extracellular matrix and fewer abilities for body circulation.^23^, ^24^ For example, the long isoform FST315 is considered as a major circulating form, whereas the short one FST288 only acts locally.^23^ Acceleron Pharma engineered a fusion protein FST288‐Fc that is capable to promote localized, rather than systemic, growth of skeletal muscle in a mice model, in which FST288 is coupled with the IgG Fc domain.^25^ Later on, Acceleron Pharma developed another follistatin fusion protein ACE‐083 with IgG Fc fused on FST291 instead of FST288 isoform. ACE‐083 treatment also showed a significant effect on local muscle hypertrophy and increased focal force generation in targeted muscle in a mouse model.^26^ In a phase I study performed on healthy postmenopausal women (NCT02257489, completed in 2015), ACE‐083 treatment significantly promoted targeted muscle growth but did not improve muscle strength.^27^ Similar to the phase I result, there is a significant increase in patient’s muscle volume, but no significant improvement on any of the functional outcomes results in early terminations of its phase II trials in patients with FSHD (NCT02927080, terminated in 2019) and CMT (NCT03124459, terminated in 2020).

Besides injection of follistatin fusion proteins, the intramuscular gene transfer of various FST isoforms to promote muscle hypertrophy has been proved in various animal models.^28^, ^29^, ^30^ FST‐344 gene therapy delivered by adeno‐associated viral vector AAV1 (rAAV1.CMV.huFollistatin 344), developed by Nationwide Children’s Hospital and Milo Biotechnology, has been tested in some small‐scale clinical trials in patients with Becker muscular dystrophy (BMD), sporadic inclusion body myositis (sIBM), and Duchenne muscular dystrophy (DMD). FST‐344 is the follistatin isoform that is cleaved to generate FST‐315. In the phase I/II trial in 15 patients with BMD and sIBM (NCT01519349, completed in 2017), one‐time treatment with direct intramuscular quadriceps injections of virus DNA (3 × 1011 vg/kg/leg or 6 × 1011 vg/kg/leg) significantly increased the 6‐minute walking distance examined in a 2‐year later functional test with no encountered adverse effects.^31^, ^32^ Corresponding to functional improvement, the treatment induces histological changes of muscle morphology, such as reduced fibrosis, reduced central nucleation, and more normal fiber size distribution with muscle hypertrophy, especially at high doses. The preliminary data of the trial with three patients with DMD (NCT02354781, completed in 2018) was recently released in 2020, however, the final evaluation report is still underway. These results show a promising application of gene therapy in treating muscle wasting disorders. Moreover, some exciting achievements in developing next‐generation vectors, by engineering newly defined muscle‐specific regulatory modules and promotors, substantially contributes to the efficiency, muscle specificity, and safety for gene therapy of muscle disorders, reviewed in ref. 33. For example, a group of potent muscle‐specific transcriptional cis‐regulatory modules (CRMs), newly identified through a genomewide mining strategy, can extensively increase transgene expression up to 400‐fold. Application of these CRMs results in significantly higher and sustained follistatin expression, which successfully leads to a robust phenotypic correction without evoking an immune response in a dystrophic mouse model.^33^

## 3 DRUG TARGETING RENIN‐ANGIOTENSIN SYSTEM

The Renin‐angiotensin system (RAS) is known for its robust effect on blood pressure and fluid homeostasis. In recent years, emerging evidence has clarified the role of RAS in promoting muscle atrophy in response to different chronic diseases, such as congestive heart failure, chronic kidney disease, and ventilator‐induced diaphragmatic wasting (comprehensively reviewed in ref. 34). Briefly, in a classical pathway, angiotensin I (AngI), cleaved from angiotensinogen by renin, is further converted to angiotensin II (AngII) by angiotensin converting enzyme (ACE). AngII binds angiotensin II type 1 receptor (AT1R) and activates the downstream PKC and/or Src pathway, resulting in the activation of NADPH oxidase II (Nox2), which upregulates the production of reactive oxygen species (ROS). Subsequently, this oxidative stress leads to muscle atrophy by accelerating protein degradation and depressing protein synthesis. In the nonclassical pathway, Ang 1‐7, generated from Ang1‐9, AngI, and AngII, binds and activates the mitochondrial assembly receptor (MASR). The activation of MASR conversely inhibits AT1R activation as well as its downstream effects (Figure 1). Moreover, a high circulating level of AngII induces high plasma levels of glucocorticoids, interleukin 6 (IL6), and serum amyloid A (SAA), inhibits the expression of IGF‐1 but promotes the expression of MSTN, which promotes skeletal muscle atrophy through AT1 receptor‐independent signaling (Figure 1).

RAS receptors not only locate on cell membranes but also locate on nuclear membranes and mitochondrial membranes. Mitochondrial RAS signaling is coupled to mitochondrial nitric oxide production and can modulate respiration. The aging‐related change in Ang receptor expression may impact mitochondrial dysfunction associated with aging.^35^

Inhibition of RAS signaling will inhibit muscle atrophy and potentially leads to a therapeutic application to treat muscle‐wasting conditions, such as sarcopenia. Currently, there are three types of drugs targeting the RAS signaling process in clinical trials: ACE inhibitors, AT1 receptor antagonists, and MASR agonists.

### 3.1 ACE inhibitors

ACE inhibitors block the production of AngII and potentially inhibit the development of sarcopenia by inhibiting AngII mediated muscle atrophy. ACE inhibitors are common drugs to treat cardiovascular diseases and prevent strokes for many years, during which their effect on promoting muscle function has been exposed. For example, elderly patients with hypertension taking ACE inhibitor drugs have a significantly slower decline in muscle strength and mobility than the ones taking other antihypertensive drugs. The muscle mass of the lower limbs is also higher than that of elderly patients taking other antihypertensive drugs.^36^, ^37^ ACE inhibitor treatment also promotes the blood supply to the muscle cells, inhibits inflammation, increases IGF‐I levels, and increases the number of mitochondria.^38^

Several currently active trials are aiming to evaluate the effect of ACE inhibitors on physical function in older people. A randomized controlled trial (RCT; EudraCT # 2014‐003455‐61; Active) in 440 patients aged 70 years and over with sarcopenia will determine the efficacy of supplementation with the amino acid leucine and/or perindopril (ACE inhibitor) to potentially improve muscle mass and function in people with sarcopenia.^39^ Another RCT (NCT01891513; completed 2017) assessed the feasibility, safety, and protocol integrity to support the conduct of a fully powered RCT to evaluate the efficacy of three antihypertensive drugs, including the ACE inhibitor perindopril, the AT1 receptor antagonist losartan, and the thiazide diuretic hydrochlorothiazide, to improve functional status in hypertensive seniors when combined with chronic exercise.^40^ A subsequent RCT (NCT03295734, currently recruiting) is under the recruiting stage, in which 213 inactive, community‐dwelling adults aged 60 years or over with hypertension and functional limitations are engaged in a 32‐week study. The trial will compare drug effects on self‐paced gait speed, exercise capacity, body mass, and composition and circulating indices of cardiovascular risk.^41^

### 3.2 Angiotensin II type I receptor antagonists

AT1 receptor antagonist blocks the activation of AT1 receptor induced by binding AngII. Like ACE inhibitors, they are often used for treating hypertension and heart failure in older adults. An AT1 receptor antagonist, losartan, improved muscle remodeling after cardiotoxin‐induced injury and protected against disuse atrophy in sarcopenia in a mouse model.^42^ In another mouse trial, losartan treatment significantly improved motilities, reduced inflammation, and reduced oxidize stress.^43^ However, losartan treatment failed to prevent mobility loss in the phase II trial (NCT02676466, completed in 2018) conducted in older adults with low‐grade inflammation and mobility limitations.^44^ Although the final evaluation has not been published yet, the newly released result of a phase II trial (NCT01989793, completed in 2016) showed no significant difference between losartan treatment and placebo control in preventing muscle strength loss associated with aging.

### 3.3 MASR agonists

MASR receptor agonist activates MASR to inhibit downstream signaling of the AT1 receptor, resulting in the attenuation of muscle atrophy. A MASR agonist, AVE 0991, slowed tumor development, reduced weight loss, improved locomotor activity, and suppressed muscle wasting in mice with cancer cachexia.^45^ Twenty‐Hydroxyecdysone (20E) is a steroid hormone essential for insect development and recently identified as a MASR agonist.^46^ BIO101, developed by Biophytis, is a pharmaceutical‐grade oral preparation of immediate‐release 20E at ≥97% purity extracted from *Cyanotis sp* plants. BIO101 is currently assessed in a phase II trial (NCT03452488; active, not recruiting) to evaluate the safety, tolerability, and efficiency in improving muscle strength in older people.

## 4 TESTOSTERONE AND SELECTIVE ANDROGEN RECEPTOR MODULATORS

### 4.1 Testosterone

As one of the sex steroids, testosterone plays an important role in the maintenance of muscle mass and function and has been extensively examined for the prevention of muscle wasting associated with aging and chronic disease.^47^ Testosterone binds to the androgen receptor (AR), which leads AR to translocate from the cytoplasm to nuclear to regulate myogenic gene expression.^48^ This interaction also intrigues a series of cellular signal transduction. In cell and mice models, testosterone treatment stimulates Akt/mTORC1 to promote protein synthesis and suppresses FoxO‐targeted gene expression to inhibit protein degradation.^49^ By suppressing the myostatin expression, testosterone treatment activates Akt/Notch signaling to promote activation and proliferation of satellite cells for muscle repairment and regeneration; it also inhibits JUN kinase‐regulated cell apoptosis.^50^

In the first testosterone trial in sarcopenia (NCT00240981, terminated in 2009), the testosterone supplementation group significantly increase maximal voluntary muscle strength in older men with low testosterone levels and mobility limitations. However, the trial was terminated due to a higher rate of adverse cardiovascular events.^51^ Later on, more clinical evidence has shown the beneficial effect of testosterone in improving muscle function. For example, testosterone treatment improved self‐reported walking ability and 6‐minute walk test distance but did not affect the rate of falls in elderly men with low testosterone in a phase III trial (NCT00799617; completed in 2014).^52^ In another phase II trial (NCT00104572; completed in 2015), the treatment improved fast gait speed at 3 and 12 months and knee strength at 12 months compared to the placebo group.^53^ However, it also leads to prostatic hyperplasia and lower urinary tract symptoms.^54^ With more side effects showing up, such as allergic reactions, thrombosis, and prostate cancer, whether the benefit overcomes the risk remains undebated.^55^, ^56^ One possible solution is to combine the testosterone treatment with other risk‐reducing treatments. For example, the risk of prostate hyperplasia can be limited by the combined treatment of testosterone and finasteride. Finasteride inhibits 5α‐reductase to converse testosterone to dihydrotestosterone (DHT), which drives the development of prostatic hyperplasia. This treatment significantly increased muscle strength and bone mineral density without causing a prostate enlargement in a phase II trial (NCT00475501; completed in 2014).^57^ Besides reducing side effects, the futural study also needs to address the concern about the persistence of beneficial effects. This issue was raised by the observation of a phase IV trial (NCT00190060; completed in 2008). Testosterone treatment for 6 months efficiently increased lean mass, isometric knee extension torque, and quality of life measures. However, these effects were lost at 6 months after the discontinuation of treatment.^58^ Longer investigation duration and dosing variation strategy will help to determine the long‐term effect of testosterone in a futural trial.

Recently, the clinical trials are more focused on the determination of the effects of testosterone combined with other treatments, such as exercise (NCT02938923, recruiting) and goserelin, a sex hormone inhibitor (NCT03054168, unknown stage). These new data will contribute to developing a new combined treatment strategy shortly when their results are reported.

### 4.2 Selective androgen receptor modulators

The selective androgen receptor modulators (SARMs) are a group of chemically synthesized small molecules that function as agonists/antagonists of AR. Similar to testosterone, the interaction of SARM and AR intrigues the translocation of the complex to nuclear. Unlike testosterone‐AR complex, different SARM‐AR complexes selectively recruit different regulatory proteins and transcriptional factors to modulate gene expression. Due to this selectivity along with the tissue‐dependent variation in AR’s expressional pattern and regulatory protein profile, SARM‐ARs display tissue‐specific signaling. Therefore, SARMs therapy can facilitate tissue‐specific benefits without off‐target side effects.^59^ SARMs studies only started in the late 20th century. With a significant anabolic activity in muscle and bone, but minimal to moderate androgenic side effects, several SARMs in preclinic studies, such as MK‐0773, GTx‐024, and GSK2881078, shows a great potential to treat muscle wasting associated conditions, including sarcopenia.

MK‐0773 has completed a phase II trial in women with mobility disabilities (NCT00529659: completed in 2009). MK‐0773 treatment for 6 months significantly increased lean body mass without evidence of androgenization. However, there is no significant improvement in muscular strength or physical performance.^60^ GTx‐024 was considered as a future star because it showed a dose‐dependent improvement in total lean body mass and physical function and was well tolerated in its phase II trial in healthy elderly men and postmenopausal women.^61^ However, it failed in the phase III trial (NCT01355497 and NCT01355484, completed in 2014) to prevent and treat muscle wasting in patients with non‐small cell lung cancer. Similar to MK‐0773, GTx‐024 treatment for 3 months significantly improved lean body mass but not physical function measured by the stair climb test.^62^, ^63^, ^64^ GSK2881078 treatment showed a dose‐dependent gain in lean body mass in a phase I trial in healthy old men and women.^65^ A phase II trial (NCT03359473, completed in 2019) was conducted to evaluate GSK2881078’s safety and effect on physical strength and function in both postmenopausal female and older male subjects with COPD and muscle weakness. Interestingly, the trial is the first one to evaluate a treatment that combined SARMs with exercise by adding a 13‐week exercise program after a 30‐day treatment of GSK2881078. The final evaluation is still underway and has not been published yet.

S42, another SARM newly developed in 2009, has shown the potential for muscle growth in an *in vitro* model (C2C12 muscle cell line). It positively impacts on muscle hypertrophy by activating Akt/mTORC1/p70S6K signaling to promote protein synthesis, and negatively impacts on muscle atrophy by suppressing the expression of MuRF1 and atrogin1 to inhibit protein degradation.^66^

## 5 GHRELIN AND ITS MIMETICS

Ghrelin is a peptide hormone secreted predominantly from the stomach. As the ligand of growth hormone (GH)‐secretagogue receptor (GHS‐R), it elicits multiple endocrine effects. It promotes growth hormone secretion, at the same time, it inhibits the production of inflammatory factors IL‐1β, IL‐6, and TNF‐α,^67^ activates mitochondria activity,^68^ thereby promoting muscle growth and improving muscle function.

Clinic studies have shown ghrelin and ghrelin mimetics significantly increase appetite and body weight, as well as have a beneficial effect in antagonizing protein breakdown and weight loss in the catabolic conditions associated with cancer cachexia, chronic heart failure (CHF), COPD, and age‐related muscle loss and frailty, comprehensively reviewed in ref. 69

For example, GH secretagogues (Capromorelin) significantly increased lean body mass and improved muscle function measured by tandem walk and stair climb tests after 2‐year treatment in healthy older adults in a phase II trial (NCT00527046, terminated in 2011).^70^ MK‐667 increased fat‐free mass without measurable change in muscle strength or function after 1 year of treatment in a preclinic trial in healthy older people.^71^ In a phase III trial (NCT01387269 and NCT01387282, completed in 2015), enrolled with 495 patients with advanced non‐small cell lung cancer, and Anamorelin treatment for 12 weeks significantly increased lean body mass, but not handgrip strength.^72^ Another phase I trial (NCT04021706, ongoing) is currently underway to determine the effect of Anamorelin on muscle and bone in patients with osteosarcopenia, which will provide more direct information for treating sarcopenia.

## 6 METFORMIN

As mentioned earlier, the drug‐induced increase of muscle mass did not drive improvement of physical function in the clinical trials of many potential sarcopenia drugs, such as MSTN inhibitors. These failures bring tremendous attention to the drugs capable of improving muscle function by mimicking the physiologic impacts of exercise. Metformin, capable of mimicking exercise‐activated activated protein kinase (AMPK) signaling, becomes one of the most attractive candidates.

Metformin is a commonly used drug for treating type II diabetes, and recently its potential on sarcopenia treatment has been explored. By activating AMPK, metformin benefit muscle hypotrophy by modulating multiple biological processes such as glucose uptake, fatty acid oxidation, protein metabolism, autophagy, and mitochondrial function.^73^, ^74^ Interestingly, metformin presents a hermetic effect on AMPK signaling with the dose‐response phenomenon, characterized by a low dose stimulation and high dose inhibition, resulting in an adaptive response to cellular metabolism variations.^75^ It can also suppress the development of sarcopenia by inhibiting NF‐kappaB mediated inflammation and oxidation response.^76^ In several model systems, metformin can extend the lifespan and improve physical performance.^77^

Metformin was hypothesized to improve or argue the exercise training effect in seniors,^78^ however, recent studies have presented a more complex scenery when combining metformin and exercise. For example, although both metformin and exercise improved insulin sensitivity in individuals with prediabetes, combined treatments did not provide additional effects.^79^ New data from a phase I trial (NCT02308228, completed in 2018) found that 14‐week treatment with 1700 mg/day of metformin blunts muscle hypertrophy in response to progressive resistance exercise training in older adults.^80^ Recently, a study in the sarcopenia mice model seems to settle the great controversy if metformin treatment improves or nullifies exercise training. It has shown a long‐term metformin treatment did not eliminate the beneficial effect given by exercise and supported the use of metformin for anti‐aging effects.^81^ More clinic trials conducted in elderly adults with prediabetes to evaluate the impacts of metformin on muscle size, strength, and physical function are currently ongoing or under data evaluation stage, including a phase I/II trial (NCT01804049, completed in 2018), a phase II trial (NCT02570672, ongoing), and a phase II trial (NCT03309007, ongoing).

## 7 CONCLUSION

The occurrence and development of sarcopenia are affected by many factors: lack of exercise and nutrition, changes in the function of the endocrine system, oxidative stress and mitochondrial dysfunction, imbalances in protein synthesis and degradation pathways, denervation of skeletal muscles, abnormal skeletal muscle repair, gut microbiome dysbiosis, and the influence of genetic factors. The complexity of this disease brings a big challenge but also plenty of opportunities for its drug development. Many drug candidates have reached clinical stages including: newly developed drugs targeting proteostasis and mitochondrial signaling through MSTN, RAS, and AMPK pathways; as well as repurposed hormonal drugs with growth‐promoting or anti‐inflammation effects, such as testosterone, insulin, Ghrelin, etc. Some of them have shown a great potential to enhance lean mass in clinic trials, but the translation of these benefits to clinically relevant improvements in muscular strength and physical performance requires further evaluation. Full consideration of the complexity by combining pharmacotherapy with other existing or emerging treatments, such as nutrition support, exercise training or exercise mimetic supplement, neural stimulation, caloric restriction, steam cell therapy, and gut–muscle axis treatment, is required to be evaluated in the future study. Besides the measurements of muscle mass, strength, and physical performance, an application of comprehensive metabolic and functional assessments in drug evaluation is also suggested to fully understand the drug’s beneficial effects on systemic health instead of limited on muscle mass.^82^ Moreover, a consensus of the conduct of clinical trials for sarcopenia has been formulated.^83^ This will improve the methodological robustness and comparability of the clinical trials, in turn, exceedingly accelerating the drug development.

## CONFLICT OF INTEREST

Nothing to declare.

## AUTHOR CONTRIBUTIONS

Yang Feike completed the collection and analysis of relevant literature and drafted the manuscript as the main writer of the review. Liu Zhijie and Chen Wei participates in the analysis and sorting of literature materials. All authors have read and approved the content of the manuscript.

## References

[1] Argilés JM, Orpí M, Busquets S, López‐Soriano FJ. Myostatin: more than just a regulator of muscle mass. Drug Discov Today. 2012;17(13–14):702‐709. 22342983 10.1016/j.drudis.2012.02.001

[2] Suh J, Lee YS. Myostatin inhibitors: panacea or predicament for musculoskeletal disorders? J Bone Metab. 2020;27(3):151‐165. 32911580 10.11005/jbm.2020.27.3.151 PMC7571243

[3] Sartori R, Gregorevic P, Sandri M. TGFbeta and BMP signaling in skeletal muscle: potential significance for muscle‐related disease. Trends Endocrinol Metab. 2014;25(9):464‐471. 25042839 10.1016/j.tem.2014.06.002

[4] Walker RG, Poggioli T, Katsimpardi L, et al. Biochemistry and biology of GDF11 and myostatin: similarities, differences, and questions for future investigation. Circ Res. 2016;118(7):1125‐1141. 27034275 10.1161/CIRCRESAHA.116.308391 PMC4818972

[5] Trendelenburg AU, Meyer A, Rohner D, et al. Myostatin reduces Akt/TORC1/p70S6K signaling, inhibiting myoblast differentiation and myotube size. Am J Physiol Cell Physiol. 2009;296(6):C1258‐C1270. 19357233 10.1152/ajpcell.00105.2009

[6] Tan LJ, Liu SL, Lei SF. Molecular genetic studies of gene identification for sarcopenia. Hum Genet. 2012;131(1):1‐31. 21706341 10.1007/s00439-011-1040-7

[7] Whittemore LA, Song K, Li X, et al. Inhibition of myostatin in adult mice increases skeletal muscle mass and strength. Biochem Biophys Res Commun. 2003;300(4):965‐971. 12559968 10.1016/s0006-291x(02)02953-4

[8] Lebrasseur NK, Schelhorn TM, Bernardo BL, et al. Myostatin inhibition enhances the effects on performance and metabolic outcomes in aged mice. J GerontolA Biol Sci Med Sci. 2009;64(9):940‐948. 10.1093/gerona/glp068 19483181

[9] Suh J, Kim NK, Lee SH, et al. GDF11 promotes osteogenesis as opposed to MSTN, and follistatin, a MSTN/GDF11 inhibitor, increases muscle mass but weakens bone. Proc Natl Acad Sci USA. 2020;117(9):4910‐4920. 32071240 10.1073/pnas.1916034117 PMC7060712

[10] Becker C, Lord SR, Studenski SA, et al. Myostatin antibody (LY2495655) in older weak fallers: a proof‐of‐concept, randomised, phase 2 trial. Lancet Diabetes Endocrinol. 2015;3(12):948‐957. 26516121 10.1016/S2213-8587(15)00298-3

[11] Wagner KR, Fleckenstein JL, Amato AA, et al. A phase I/IItrial of MYO‐029 in adult subjects with muscular dystrophy. Ann Neurol. 2008;63:561‐571. 18335515 10.1002/ana.21338

[12] Skelet Muscle. IMB0901 inhibits muscle atrophy induced by cancer cachexia through MSTN signaling pathway. 2019;9(1):8. 10.1186/s13395-019-0193-2 30922397 PMC6437903

[13] Morvan F, Rondeau JM, Zou C, et al. Blockade of activin type II receptors with a dual anti‐ActRIIA/IIB antibody is critical to promote maximal skeletal muscle hypertrophy. Proc Natl Acad Sci USA. 2017;114:12448‐12453. 29109273 10.1073/pnas.1707925114 PMC5703284

[14] Lach‐Trifilieff E, Minetti GC, Sheppard K, et al. An antibody blocking activin type II receptors induces strong skeletal muscle hypertrophy and protects from atrophy. Mol Cell Biol. 2014;34:606‐618. 24298022 10.1128/MCB.01307-13 PMC3911487

[15] Rooks D, Praestgaard J, Hariry S, et al. Treatment of sarcopenia with bimagrumab: results from a phase II, randomized, controlled, proof‐of‐concept study. J Am Geriatr Soc. 2017;65:1988‐1995. 28653345 10.1111/jgs.14927

[16] Rooks D, Swan T, Goswami B, et al. Bimagrumab vs optimized standard of care for treatment of sarcopenia in community‐dwelling older adults: a randomized clinical trial. JAMA Netw Open. 2020;3:e2020836. 10.1001/jamanetworkopen.2020.20836 33074327 PMC7573681

[17] Hanna MG, Badrising UA, Benveniste O, et al. Safety and efficacy of intravenous bimagrumab in inclusion body myositis (RESILIENT): a randomised, double‐blind, placebo‐controlled phase 2b trial. Lancet Neurol. 2019;18:834‐844. 31397289 10.1016/S1474-4422(19)30200-5

[18] Polkey MI, Praestgaard J, Berwick A, et al. Activin type II receptor blockade for treatment of muscle depletion in chronic obstructive pulmonary disease. A randomized trial. Am J Respir Crit Care Med. 2019;199:313‐320. 30095981 10.1164/rccm.201802-0286OC PMC6363975

[19] Cadena SM, Tomkinson KN, Monnell TE, et al. Administration of a soluble activin type IIB receptor promotes skeletal muscle growth independent of fiber type. J Appl Physiol. 2010;109:635‐642. 20466801 10.1152/japplphysiol.00866.2009 PMC2944638

[20] Attie KM, Borgstein NG, Yang Y, et al. A single ascending‐dose study of muscle regulator ACE‐031 in healthy volunteers. Muscle Nerve. 2013;47:416‐423. 23169607 10.1002/mus.23539

[21] Campbell C, McMillan HJ, Mah JK, et al. Myostatin inhibitor ACE‐031 treatment of ambulatory boys with Duchenne muscular dystrophy: Results of a randomized, placebo‐controlled clinical trial. Muscle Nerve. 2017;55:458‐464. 27462804 10.1002/mus.25268

[22] Tauer JP, Rauch F. Novel ActRIIB ligand trap increases muscle mass and improves bone geometry in a mouse model of severe osteogenesis imperfecta. Bone. 2019;128:115036. 31419601 10.1016/j.bone.2019.115036

[23] Schneyer AL, Wang Q, Sidis Y, Sluss PM. Differential distribution of follistatin isoforms: application of a new FS315‐specific immunoassay. J Clin Endocrinol Metab. 2004;89:5067‐5075. 15472207 10.1210/jc.2004-0162

[24] Sidis Y, et al. Biological activity of follistatin isoforms and follistatin‐like‐3 is dependent on differential cell surface binding and specificity for activin, myostatin, and bone morphogenetic proteins. Endocrinology. 2006;147:3586‐3597. 16627583 10.1210/en.2006-0089

[25] Castonguay R, Lachey J, Wallner S, et al. Intramuscular administration of FST288‐Fc in mice induced robust, dose‐dependent growth of the targeted muscle but not of surrounding or contralateral muscles. J Pharmacol Exp Ther. 2019;368(3):435‐445. 30563942 10.1124/jpet.118.252304

[26] Pearsall RS, Davies MV, Cannell M, et al. Follistatin‐based ligand trap ACE‐083 induces localized hypertrophy of skeletal muscle with functional improvement in models of neuromuscular disease. Sci Rep. 2019;9(1):11392. 31388039 10.1038/s41598-019-47818-w PMC6684588

[27] Glasser CE, Gartner MR, Wilson D, et al. Locally acting ACE‐083 increases muscle volume in healthy volunteers. Muscle Nerve. 2018;57(6):921‐926. 29486514 10.1002/mus.26113 PMC5969095

[28] Haidet AM, Rizo L, Handy C,, et al. Long‐term enhancement of skeletal muscle mass and strength by single gene administration of myostatin inhibitors. Proc Natl Acad Sci USA. 2008;105:4318‐4322. 10.1073/pnas.0709144105 18334646 PMC2393740

[29] Kota J, Handy CR, Haidet AM, et al. Follistatin gene delivery enhances muscle growth and strength in nonhuman primates. Sci Transl Med. 2009;1(6):6ra15. 10.1126/scitranslmed.3000112 PMC2852878 20368179

[30] Giesige CR, Wallace LM, Heller KN, et al. AAV‐mediated follistatin gene therapy improves functional outcomes in the TIC‐DUX4 mouse model of FSHD. JCI Insight. 2018;3(22):e123538. 10.1172/jci.insight.123538 PMC6302942 30429376

[31] Mendell JR, Sahenk Z, Malik V, et al. A phase 1/2a follistatin gene therapy trial for becker muscular dystrophy. Mol Ther. 2015;23:192‐201. 25322757 10.1038/mt.2014.200 PMC4426808

[32] Mendell JR, Sahenk Z, Al‐Zaidy S, et al. Follistatin gene therapy for sporadic inclusion body myositis improves functional outcomes. Mol Ther. 2017;25:870‐879. 28279643 10.1016/j.ymthe.2017.02.015 PMC5383643

[33] Sarcar S, Tulalamba W, Rincon MY, et al. Next‐generation muscle‐directed gene therapy by in silico vector design. Nat Commun. 2019;10:492. 30700722 10.1038/s41467-018-08283-7 PMC6353880

[34] Powers SK, Morton AB, Hyatt H, Hinkley MJ. The renin‐angiotensin system and skeletal muscle. Exerc Sport Sci Rev. 2018;46(4):205‐214. 30001274 10.1249/JES.0000000000000158 PMC6673677

[35] Abadir PM, Foster DB, Crow M, et al. Identification and characterization of a functional mitochondrial angiotensin system. Proc Natl Acad Sci USA. 2011;108(36):14849‐14854. 21852574 10.1073/pnas.1101507108 PMC3169127

[36] Onder G, Penninx BW, Balkrishnan R, et al. Relation between use of angiotensin‐converting enzyme inhibitors and muscle strength and physical function in older women: an observational study. Lancet. 2002;359(9310):926‐930. 11918911 10.1016/s0140-6736(02)08024-8

[37] Hutcheon SD, Gillespie ND, Crombie IK, et al. Perindopril improves six minute walking distance in older patients with left ventricular systolic dysfunction: a randomised double blind placebo controlled trial. Heart. 2002;88(4):373‐377. 12231595 10.1136/heart.88.4.373 PMC1767356

[38] Maggio M, Ceda GP, Lauretani F, et al. Relation of angiotensin converting enzyme inhibitor treatment to insulin‐like growth factor‐1 serum levels in subjects > 65 years of age (the InCHIANTI study). Am J Cardiol. 2006;97(10):1525‐1529. 16679098 10.1016/j.amjcard.2005.11.089 PMC2646084

[39] Band MM, Sumukadas D, Struthers AD, et al. Leucine and ACE inhibitors as therapies for sarcopenia (LACE trial): study protocol for a randomised controlled trial. Trials. 2018;19(1):6. 29301558 10.1186/s13063-017-2390-9 PMC5753568

[40] Baptista LC, Jaeger BC, Anton SD, et al. Multimodal intervention to improve functional status in hypertensive older adults: a pilot randomized controlled trial. J Clin Med. 2019;8:2. 10.3390/jcm8020196 PMC6406861 30736317

[41] Harper SA, Baptista LC, Roberts LM, et al. Angiotensin converting enzyme inhibitors combined with exercise for hypertensive seniors (the ACES trial): study protocol of a randomized controlled trial. Front Med (Lausanne). 2020;6:327. 32039215 10.3389/fmed.2019.00327 PMC6988302

[42] Burks TN, Andres‐Mateos E, Marx R, et al. Losartan restores skeletal muscle remodeling and protects against disuse atrophy in sarcopenia. Sci Transl Med. 2011;3(82):82ra37. 10.1126/scitranslmed.3002227 PMC3140459 21562229

[43] Lin CH, Yang H, Xue QL, et al. Losartan improves measures of activity, inflammation, and oxidative stress in older mice. Exp Gerontol. 2014;58:174‐178. 25077714 10.1016/j.exger.2014.07.017 PMC4252828

[44] Pahor M, Anton SD, Beavers DP, et al. Effect of losartan and fish oil on plasma IL‐6 and mobility in older persons. The ENRGISE Pilot randomized clinical trial. J Gerontol A Biol Sci Med Sci. 2019;74(10):1612‐1619. 30541065 10.1093/gerona/gly277 PMC6748815

[45] Murphy KT, Hossain MI, Swiderski K, et al. Mas receptor activation slows tumor growth and attenuates muscle wasting in cancer. Cancer Res. 2019;79(4):706‐719. 30420474 10.1158/0008-5472.CAN-18-1207 PMC6377850

[46] Lafont R, Raynal S, Serova M, et al. 20‐Hydroxyecdysone activates the protective arm of the renin angiotensin system via Mas receptor. BioRxiv. 2020. https://doi.org/10.1101/2020.04.08.032607 10.1530/JME-21-0033 34825653

[47] Saad F, Rohrig G, von Haehling S, et al. Testosterone deficiency and testosterone treatment in older men. Gerontology. 2017;63(2):144‐156. 27855417 10.1159/000452499

[48] Morley JE. Hormones and sarcopenia. Curr Pharm Des. 2017;23(30):4484‐4492. 27881060 10.2174/1381612823666161123150032

[49] White JP, Gao S, Puppa MJ, et al. Testosterone regulation of Akt/mTORC1/FoxO3a signaling in skeletal muscle. Mol Cell Endocrinol. 2013;365(2):174‐186. 23116773 10.1016/j.mce.2012.10.019 PMC3529800

[50] Kovacheva EL, Hikim AP, Shen R, et al. Testosterone supplementation reverses sarcopenia in aging through regulation of myostatin, c‐Jun NH2‐terminal kinase, Notch, and Akt signaling pathways. Endocrinology. 2010;151(2):628‐638. 20022929 10.1210/en.2009-1177 PMC2817626

[51] LeBrasseur NK, Lajevardi N, Miciek R, et al. Effects of testosterone therapy on muscle performance and physical function in older men with mobility limitations (The TOM Trial): design and methods. Contemp Clin Trials. 2009;30(2):133‐140. 18996225 10.1016/j.cct.2008.10.005 PMC3031114

[52] Bhasin S, Ellenberg SS, Storer TW, et al. Effect of testosterone replacement on measures of mobility in older men with mobility limitation and low testosterone concentrations: secondary analyses of the testosterone trials. Lancet Diabetes Endocrinol. 2018;6(11):879‐890. 30366567 10.1016/S2213-8587(18)30171-2 PMC6816466

[53] Dias JP, Veldhuis JD, Carlson O, et al. Effects of transdermal testosterone gel or an aromatase inhibitor on serum concentration and pulsatility of growth hormone in older men with age‐related low testosterone. Metabolism. 2017;69(4):143‐147. 28285644 10.1016/j.metabol.2017.01.025 PMC5950718

[54] Dias JP, Melvin D, Shardell M, et al. Effects of Transdermal testosterone gel or an aromatase inhibitor on prostate volume in older men. J Clin Endocrinol Metab. 2016;101(4):1865‐1871. 26950683 10.1210/jc.2016-1111 PMC4880169

[55] Lynch GS. Update on emerging drugs for sarcopenia ‐ age‐related muscle wasting. Expert OpinEmerg Drugs. 2008;13(4):655‐673. 10.1517/14728210802544476 19046133

[56] Grech A, Breck J, Heidelbaugh J. Adverse effects of testosterone replacement therapy: an update on the evidence and controversy. Ther Adv Drug Saf. 2014;5(5):190‐200. 25360240 10.1177/2042098614548680 PMC4212439

[57] Borst SE, Yarrow JF, Conover CF, et al. Musculoskeletal and prostate effects of combined testosterone and finasteride administration in older hypogonadal men: a randomized, controlled trial. Am J Physiol Endocrinol Metab. 2014;306:E433‐442. 24326421 10.1152/ajpendo.00592.2013 PMC4073894

[58] O’Connell MD, Roberts SA, Srinivas‐Shankar U, et al. Do the effects of testosterone on muscle strength, physical function, body composition, and quality of life persist six months after treatment in intermediate‐frail and frail elderly men? J Clin Endocrinol Metab. 2011;96(2):454‐458. 21084399 10.1210/jc.2010-1167

[59] Christiansen AR, Lipshultz JI, Hotaling JM. Selective androgen receptor modulators: the future of androgen therapy? Transl Androl Urol. 2020;9(Suppl 2):S135‐S148. 32257854 10.21037/tau.2019.11.02 PMC7108998

[60] PapanicolaouDA, AtherSN, ZhuH, et al. A phase IIA randomized, placebo‐controlled clinical trial to study the efficacy and safety of the selective androgen receptor modulator (SARM), MK‐0773 in female participants with sarcopenia. J Nutr Health Aging. 2013;17(6):533‐543.2373255010.1007/s12603-013-0335-x

[61] Dalton JT, Barnette KG, Bohl CE, et al. The selective androgen receptor modulator GTx‐024 (enobosarm) improves lean body mass and physical function in healthy elderly men and postmenopausal women: results of a double‐blind, placebo‐controlled phase II trial. J Cachexia Sarcopenia Muscle. 2011;2(3):153‐161. 22031847 10.1007/s13539-011-0034-6 PMC3177038

[62] GTx Reports Results for Enobosarm POWER Trials for the Prevention and Treatment of Muscle Wasting in Patients with Non‐Small Cell Lung Cancer. 2013. Available online: https://www.businesswire.com/news/home/20130819005378/en/GTx‐Reports‐Results‐Enobosarm‐POWER‐Trials‐Prevention

[63] Crawford J, Prado CM, Johnston MA, et al. Study design and rationale for the phase 3 clinical development program of enobosarm, a selective androgen receptor modulator, for the prevention and treatment of muscle wasting in cancer patients (POWER Trials). Curr Oncol Rep. 2016;18(6):37. 27138015 10.1007/s11912-016-0522-0 PMC4853438

[64] Dalton JT. The long and winding road for selective androgen receptor modulators. Br J Clin Pharmacol. 2017;83(10):2131‐2133. 28621446 10.1111/bcp.13345 PMC5595949

[65] Neil D, Clark RV, Magee M, et al. GSK2881078, a SARM, produces dose‐dependent increases in lean mass in healthy older men and women. J Clin Endocrinol Metab. 2018;103(9):3215‐3224. 29982690 10.1210/jc.2017-02644

[66] Muta Y, Tanaka T, Hamaguchi Y, et al. Selective androgen receptor modulator, S42 has anabolic and anti‐catabolic effects on cultured myotubes. Biochem Biophys Rep. 2019;17:177‐181. 30705972 10.1016/j.bbrep.2019.01.006 PMC6348734

[67] Dixit VD, Schaffer EM, Pyle RS, et al. Ghrelin inhibits leptin‐ and activation‐induced proinflammatory cytokine expression by human monocytes and T cells. J Clin Invest. 2004;114(1):57‐66. 15232612 10.1172/JCI21134 PMC437970

[68] Tamaki M, Miyashita K, Hagiwara A, et al. Ghrelin treatment improves physical decline in sarcopenia model mice through muscular enhancement and mitochondrial activation. Endocr J. 2017;64(Suppl.):S47‐S51. 28652544 10.1507/endocrj.64.S47

[69] Nass R, Gaylinn BD, Thorner MO. The ghrelin axis in disease: potential therapeutic indications. Mol Cell Endocrinol. 2011;340(1):106‐110. 21356273 10.1016/j.mce.2011.02.010 PMC3114265

[70] White HK, Petrie CD, Landschulz W, et al. Effects of an oral growth hormone secretagogue in older adults. Capromorelin Study Group. J Clin Endocrinol Metab. 2009;94(4):1198‐1206. 19174493 10.1210/jc.2008-0632

[71] Nass R, Pezzoli SS, Oliveri MC, et al. Effects of an oral ghrelin mimetic on body composition and clinical outcomes in healthy older adults: a randomized trial. Ann Intern Med. 2008;149(9):601‐611. 18981485 10.7326/0003-4819-149-9-200811040-00003 PMC2757071

[72] Temel JS, Abernethy AP, Currow DC, et al. Anamorelin in patients with non‐small‐cell lung cancer and cachexia (ROMANA 1 and ROMANA 2): results from two randomized, double‐blind, phase 3 trials. Lancet Oncol. 2016;17(4):519‐531. 26906526 10.1016/S1470-2045(15)00558-6

[73] Zhou G, Myers R, Li Y, et al. Role of AMP‐activated protein kinase in mechanism of metformin action. J Clin Invest. 2001;108(8):1167‐1174. 11602624 10.1172/JCI13505 PMC209533

[74] Kjobsted R, Hingst JR, Fentz J, et al. AMPK in skeletal muscle function and metabolism. Faseb J. 2018;32(4):1741‐1777. 29242278 10.1096/fj.201700442R PMC5945561

[75] Panfoli I, Puddu A, Bertola N, et al. The hormetic effect of metformin: “Less Is More”? Int J Mol Sci. 2021;22(12):6297. 34208371 10.3390/ijms22126297 PMC8231127

[76] Kanigur Sultuybek G, Soydas T, Yenmis G. NF‐kappaB as the mediator of metformin’s effect on ageing and ageing‐related diseases. Clin Exp Pharmacol Physiol. 2019;46(5):413‐422. 30754072 10.1111/1440-1681.13073

[77] Kulkarni AS, Brutsaert EF, Anghel V, et al. Metformin regulates metabolic and nonmetabolic pathways in skeletal muscle and subcutaneous adipose tissues of older adults. Aging Cell. 2018;17:e12723. 10.1111/acel.12723 PMC5847877 29383869

[78] Long DE, Peck BD, Martz JL, et al. Metformin to Augment Strength Training Effective Response in Seniors (MASTERS): study protocol for a randomized controlled trial. Trials. 2017;18:192. 28441958 10.1186/s13063-017-1932-5 PMC5405504

[79] Malin SK, Gerber R, Chipkin SR, et al. Independent and combined effects of exercise training and metformin on insulin sensitivity in individuals with prediabetes. Diabetes Care. 2012;35(1):131‐136. 22040838 10.2337/dc11-0925 PMC3241331

[80] Walton RG, Dungan CM, Long DE, et al. Metformin blunts muscle hypertrophy in response to progressive resistance exercise training in older adults: a randomized, double‐blind, placebo‐controlled, multicenter trial: The MASTERS trial. Aging Cell. 2019;18(6):e13039. 31557380 10.1111/acel.13039 PMC6826125

[81] Hernández‐Álvarez D, Mena‐Montes B, Toledo‐Pérez R, et al. Long‐term moderate exercise combined with metformin treatment induces an hormetic response that prevents strength and muscle mass loss in old female wistar rats. Oxid Med Cell Longev. 2019;2019:3428543. 31814870 10.1155/2019/3428543 PMC6877950

[82] Hardee JP, Lynch GS. Current pharmacotherapies for sarcopenia. Expert Opin Pharmacother. 2019;20(13):1645‐1657. 31120352 10.1080/14656566.2019.1622093

[83] Reginster JY, Beaudart C, Al‐Daghri N. Update on the ESCEO recommendation for the conduct of clinical trials for drugs aiming at the treatment of sarcopenia in older adults. Aging Clin Exp Res. 2021;33(1):3‐17. 32737844 10.1007/s40520-020-01663-4 PMC7897619

